# Supplementary material for: Serum Metabolomic Alteration in Rats with Osteoarthritis Treated with Palm Tocotrienol-Rich Fraction Alone or in Combination with Glucosamine Sulphate
Source: Life (Basel). 2023 Dec 15;13(12):2343. doi: 10.3390/life13122343 (PMC10744932; doi:10.3390/life13122343)
Supplement: Supplementary file 1 [file life-13-02343-s001.zip › S1. OC-SC.pdf]

Table S1: List of biochemical pathways (MetaboAnalyst) identified for **OC vs SC**

Pathway	Match status	p-value	Impact
Aminoacyl-tRNA biosynthesis	6/48	1.50E-05	0.00
Valine, leucine and isoleucine biosynthesis	3/8	9.12E-05	0.00
Phenylalanine metabolism	2/12	9.20E-03*	0.36#
Arginine and proline metabolism	3/38	1.08E-02*	0.17#
Lysine degradation	2/25	3.79E-02	0.00
Glutathione metabolism	2/28	4.67E-02	0.03
Phenylalanine, tyrosine and tryptophan biosynthesis	1/4	4.95E-02*	0.50#
Synthesis and degradation of ketone bodies	1/5	6.15E-02	0.00
Glycine, serine and threonine metabolism	2/34	6.63E-02	0.02
Valine, leucine and isoleucine degradation	2/40	8.82E-02	0.00
Taurine and hypotaurine metabolism	1/8	9.66E-02	0.43
Primary bile acid biosynthesis	2/46	1.12E-01	0.02
Arginine biosynthesis	1/14	1.63E-01	0.08
Butanoate metabolism	1/15	1.74E-01	0.00
beta-Alanine metabolism	1/21	2.35E-01	0.00
Glycerophospholipid metabolism	1/36	3.70E-01	0.03
Fatty acid degradation	1/39	3.94E-01	0.00
Pyrimidine metabolism	1/39	3.94E-01	0.01
Purine metabolism	1/66	5.75E-01	0.02
Steroid hormone biosynthesis	1/77	6.33E-01	0.03

*p-value <0.05; and #impact > 0.1 is regarded as significant
