# Supplementary material for: Serum Metabolomic Alteration in Rats with Osteoarthritis Treated with Palm Tocotrienol-Rich Fraction Alone or in Combination with Glucosamine Sulphate
Source: Life (Basel). 2023 Dec 15;13(12):2343. doi: 10.3390/life13122343 (PMC10744932; doi:10.3390/life13122343)
Supplement: Supplementary file 1 [file life-13-02343-s001.zip › S2. OG-OC.pdf]

Table S2: List of biochemical pathways (MetaboAnalyst) identified for **OG vs OC**

Pathway	Match status	p-value	Impact
Aminoacyl-tRNA biosynthesis	5/48	6.24E-05	0.00
Sphingolipid metabolism	2/21	1.74E-02	0.07
Glutathione metabolism	2/28	3.00E-02	0.03
Phenylalanine, tyrosine and tryptophan biosynthesis	1/4	3.92E-02*	0.50#
Synthesis and degradation of ketone bodies	1/5	4.88E-02	0.00
Arginine and proline metabolism	2/38	5.28E-02	0.11
D-Glutamine and D-glutamate metabolism	1/6	5.83E-02	0.00
Nitrogen metabolism	1/6	5.83E-02	0.00
Tryptophan metabolism	2/41	6.05E-02	0.24
Ubiquinone and other terpenoid-quinone biosynthesis	1/9	8.62E-02	0.00
Phenylalanine metabolism	1/12	1.13E-01	0.00
Arginine biosynthesis	1/14	1.31E-01	0.00
Butanoate metabolism	1/15	1.40E-01	0.00
beta-Alanine metabolism	1/21	1.90E-01	0.00
Lysine degradation	1/25	2.23E-01	0.00
Alanine, aspartate and glutamate metabolism	1/28	2.46E-01	0.11
Glyoxylate and dicarboxylate metabolism	1/32	2.76E-01	0.00
Cysteine and methionine metabolism	1/33	2.83E-01	0.10
Glycerophospholipid metabolism	1/36	3.05E-01	0.00
Fatty acid degradation	1/39	3.26E-01	0.00
Pyrimidine metabolism	1/39	3.26E-01	0.00
Tyrosine metabolism	1/42	3.47E-01	0.14
Primary bile acid biosynthesis	1/46	3.73E-01	0.02
Purine metabolism	1/66	4.90E-01	0.00

*p-value <0.05; and #impact > 0.1 is regarded as significant
