# Supplementary material for: Serum Metabolomic Alteration in Rats with Osteoarthritis Treated with Palm Tocotrienol-Rich Fraction Alone or in Combination with Glucosamine Sulphate
Source: Life (Basel). 2023 Dec 15;13(12):2343. doi: 10.3390/life13122343 (PMC10744932; doi:10.3390/life13122343)
Supplement: Supplementary file 1 [file life-13-02343-s001.zip › S3. OT-OC.pdf]

Table S3: List of biochemical pathways (MetaboAnalyst) identified for **OT vs OC**

Pathway	Match status	p-value	Impact
Arginine and proline metabolism	3/38	0.00*	0.12#
Glutathione metabolism	2/28	0.01	0.03
Synthesis and degradation of ketone bodies	1/5	0.03	0.00
Aminoacyl-tRNA biosynthesis	2/48	0.03	0.00
Butanoate metabolism	1/15	0.09	0.00
beta-Alanine metabolism	1/21	0.12	0.00
Sphingolipid metabolism	1/21	0.12	0.04
Lysine degradation	1/25	0.14	0.00
Glycine, serine and threonine metabolism	1/34	0.19	0.00
Tryptophan metabolism	1/41	0.22	0.14
Purine metabolism	1/66	0.33	0.02

*p-value <0.05; and #impact > 0.1 is regarded as significant
