# Supplementary material for: Serum Metabolomic Alteration in Rats with Osteoarthritis Treated with Palm Tocotrienol-Rich Fraction Alone or in Combination with Glucosamine Sulphate
Source: Life (Basel). 2023 Dec 15;13(12):2343. doi: 10.3390/life13122343 (PMC10744932; doi:10.3390/life13122343)
Supplement: Supplementary file 1 [file life-13-02343-s001.zip › S4. OGT-OC.pdf]

Table S4: List of biochemical pathways (MetaboAnalyst) identified for **OGT vs OC**

Pathway	Match status	p-value	Impact
Phenylalanine, tyrosine and tryptophan biosynthesis	2/4	0.00*	1.00#
Aminoacyl-tRNA biosynthesis	4/48	0.00	0.00
Phenylalanine metabolism	2/12	0.00*	0.36#
Tryptophan metabolism	2/41	0.04*	0.24#
Ubiquinone and other terpenoid-quinone biosynthesis	1/9	0.07	0.00
Pantothenate and CoA biosynthesis	1/19	0.14	0.01
Sphingolipid metabolism	1/21	0.16	0.00
Lysine degradation	1/25	0.18	0.00
Glutathione metabolism	1/28	0.20	0.03
Cysteine and methionine metabolism	1/33	0.23	0.02
Glycerophospholipid metabolism	1/36	0.25	0.00
Arginine and proline metabolism	1/38	0.26	0.08
Fatty acid degradation	1/39	0.27	0.00
Tyrosine metabolism	1/42	0.29	0.14

*p-value <0.05; and #impact > 0.1 is regarded as significant.
