# Supplementary material for: Serum Metabolomic Alteration in Rats with Osteoarthritis Treated with Palm Tocotrienol-Rich Fraction Alone or in Combination with Glucosamine Sulphate
Source: Life (Basel). 2023 Dec 15;13(12):2343. doi: 10.3390/life13122343 (PMC10744932; doi:10.3390/life13122343)
Supplement: Supplementary file 1 [file life-13-02343-s001.zip › S5. OT-OG.pdf]

Table S5: List of biochemical pathways (MetaboAnalyst) identified for **OT vs OG**

Pathway	Match status	p-value	Impact
Lysine degradation	2/25	0.00	0.00
D-Glutamine and D-glutamate metabolism	1/6	0.02	0.00
Nitrogen metabolism	1/6	0.02	0.00
Arginine biosynthesis	1/14	0.05	0.00
Sphingolipid metabolism	1/21	0.08	0.02
Glutathione metabolism	1/28	0.11	0.01
Alanine, aspartate and glutamate metabolism	1/28	0.11	0.11
Glyoxylate and dicarboxylate metabolism	1/32	0.12	0.00
Glycine, serine and threonine metabolism	1/34	0.13	0.00
Arginine and proline metabolism	1/38	0.14	0.01
Pyrimidine metabolism	1/39	0.15	0.00
Aminoacyl-tRNA biosynthesis	1/48	0.18	0.00
Purine metabolism	1/66	0.24	0.00
