# Supplementary material for: Serum Metabolomic Alteration in Rats with Osteoarthritis Treated with Palm Tocotrienol-Rich Fraction Alone or in Combination with Glucosamine Sulphate
Source: Life (Basel). 2023 Dec 15;13(12):2343. doi: 10.3390/life13122343 (PMC10744932; doi:10.3390/life13122343)
Supplement: Supplementary file 1 [file life-13-02343-s001.zip › S6. OGT-OG.pdf]

Table S6: List of biochemical pathways (MetaboAnalyst) identified for **OGT vs OG**

Pathway	Match status	p-value	Impact
Aminoacyl-tRNA biosynthesis	3/48	0.00	0.00
Phenylalanine, tyrosine and tryptophan biosynthesis	1/4	0.02*	0.50#
D-Glutamine and D-glutamate metabolism	1/6	0.02	0.00
Nitrogen metabolism	1/6	0.02	0.00
Ubiquinone and other terpenoid-quinone biosynthesis	1/9	0.04	0.00
Phenylalanine metabolism	1/12	0.05	0.00
Arginine biosynthesis	1/14	0.05	0.00
Histidine metabolism	1/16	0.06	0.22
beta-Alanine metabolism	1/21	0.08	0.00
Sphingolipid metabolism	1/21	0.08	0.04
Glutathione metabolism	1/28	0.11	0.01
Alanine, aspartate and glutamate metabolism	1/28	0.11	0.11
Glyoxylate and dicarboxylate metabolism	1/32	0.12	0.00
Cysteine and methionine metabolism	1/33	0.12	0.02
Pyrimidine metabolism	1/39	0.15	0.00
Tyrosine metabolism	1/42	0.16	0.14
Purine metabolism	1/66	0.24	0.00

*p-value <0.05; and #impact > 0.1 is regarded as significant
