# Supplementary material for: Serum Metabolomic Alteration in Rats with Osteoarthritis Treated with Palm Tocotrienol-Rich Fraction Alone or in Combination with Glucosamine Sulphate
Source: Life (Basel). 2023 Dec 15;13(12):2343. doi: 10.3390/life13122343 (PMC10744932; doi:10.3390/life13122343)
Supplement: Supplementary file 1 [file life-13-02343-s001.zip › S7. OGT-OT.pdf]

pTable S7: List of biochemical pathways (MetaboAnalyst) identified for **OGT vs OT**

Pathway	Match status	p-value	Impact
Lysine degradation	2/25	0.00	0.00
Phenylalanine, tyrosine and tryptophan biosynthesis	1/4	0.02*	0.50#
Synthesis and degradation of ketone bodies	1/5	0.02	0.00
Ubiquinone and other terpenoid-quinone biosynthesis	1/9	0.04	0.00
Phenylalanine metabolism	1/12	0.05	0.00
Butanoate metabolism	1/15	0.06	0.00
Cysteine and methionine metabolism	1/33	0.12	0.02
Glycine, serine and threonine metabolism	1/34	0.13	0.00
Arginine and proline metabolism	1/38	0.14	0.01
Tyrosine metabolism	1/42	0.16	0.14
Aminoacyl-tRNA biosynthesis	1/48	0.18	0.00

*p-value <0.05; and #impact > 0.1 is regarded as significant
